# A nanocomposite of NiFe_2_O_4_–PANI as a duo active electrocatalyst toward the sensitive colorimetric and electrochemical sensing of ascorbic acid†

**DOI:** 10.1039/d0na00283f

**Published:** 2020-06-22

**Authors:** D. Navadeepthy, M. Thangapandian, C. Viswanathan, N. Ponpandian

**Affiliations:** Department of Nanoscience and Technology, Bharathiar University Coimbatore 641046 India ponpandian@buc.edu.in +91-422-2422397 +91-422-2426-421

## Abstract

A non-enzymatic ascorbic acid sensor using a nickel ferrite/PANI (NF–PANI) nanocomposite and based on colorimetric and electrochemical sensing methods was investigated in this study. The nanocomposite was prepared by an *in situ* polymerization and utilized as an electrocatalyst to sense ascorbic acid (AA) through the peroxidase mimic sensing of H_2_O_2_ in the presence of 3,5,3,5-tetramethylbenzidine (TMB) as a coloring agent. It was also utilized to detect AA present in real samples prepared from fruit extracts, commercial beverages, and vitamin-C tablets. The limit of detection (LoD) for AA sensing by the peroxidase mimic method was found to be 232 nM. The relative standard deviation (RSD) calculated for analysis of the real samples analysis ranged from 1.7–3.2%. Similarly, the electrochemical sensing of AA by NF–PANI was examined by cyclic voltammetric, chronoamperometric, and differential pulse voltammetric analyses. The LoD for the electrochemical method applied to AA sensing was 423 nM. The nanocomposite functioned as an effective electrocatalytic sensing agent in both methods to selectively detect AA due to the combined effect of NF and PANI. Thus, it was shown that the nanocomposites could be utilized for the laboratory-based detection of AA by various methods and could give rapid results.

## Abstract 2

A non-enzymatic, duo-active sensor using nickel ferrite/PANI (NF–PANI) nanocomposite based on peroxidase mimic and electrochemical methods for sensitive and selective detection of ascorbic acid.

## Graphical abstract

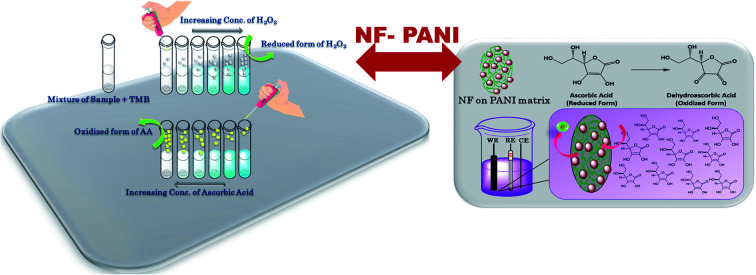

## 1. Introduction

Ascorbic acid (AA), one of the soluble vitamins present in the human body, is widely used as a preservative in the food, cosmetic, and pharmaceutical industries.^1,2^ It plays a vital role in various physiological and biochemical processes, and its deficiency can affect the metabolism and cause serious illness in the human body. The ideal range of AA in human serum is 50–70 μM. An imbalance in the level of AA may induce variation in the production of ROS and antioxidants which leads to tissue damage and related diseases.^3^ There is an urgent need to design a reliable and highly sensitive sensor for the on-site and rapid detection of AA for healthcare and food quality and security. A number of analytical techniques are employed for the detection of AA, such as liquid chromatography, spectrophotometry, fluorescence, chemiluminescence, and electrochemical methods,^4^ but even though they are prominently used and have demonstrated low detection limits with good accuracy, they are time-consuming, expensive, and require skilled personnel to be performed. Among the far-ranging analytical techniques, peroxidase mimic colorimetric sensing and electrochemical methods have attracted much attention. Colorimetric sensing strategies show results that are visible to the naked-eye and are not primarily dependent on the instrument utilized.^5^ Similarly, electrochemical methods are well established and known to have high accuracy in terms of both selectivity and sensitivity. This provides for ease of analysis, which is required in field tests to meet laboratory purposes.^6^

It is well-known that uric acid (UA) and dopamine co-exist along with AA in biofluids. Both UA and dopamine represent potential interfering species in the electrochemical detection of AA as they undergo oxidation at a potential close to that of AA. Similarly, the oxidative products from the interfering compounds may further oxidize AA, which can lead to an inaccurate analysis. Hence, it is necessary to develop a selective and sensitive method for the determination of AA.^7^

Recently, nanomaterial-based enzyme mimics have attracted much attention owing to their advantages over natural enzymes, such as easy preparation procedure, excellent stability, low cost, and high catalytic activity. In this context, tremendous efforts have been carried out to develop a nanozyme using various nanomaterials and composites with metals,^8^ metal oxides,^9^ carbon based materials,^10–12^ polymers^13^ and polymer nanocomposites^14^ and layered hydroxides^15,16^ are studied. However, they show poor catalytic activity, owing to their decreased specific surface area and aggregation in solution. Peroxidase, a natural enzyme, is conventionally used as a catalyzing agent and though it possesses excellent oxidizability, it has some inherent drawbacks, such as easy denaturation, easily digestible by protease, and expensive preparation and purification are needed.

Recently, magnetic nanoparticles have been widely employed in solution-based sensing applications since they are easily separable after the process.^17,18^ In particular, ferrites, such as ZnFe_2_O_4_,^19,20^ CoFe_2_O_4_,^21^ and MnFe_2_O_4_ (ref. 22) have been investigated and reported to be excellent peroxidase mimicking agents. The Fe^3+^ ions in the ferrite structure play a vital role in the peroxidase mimic property since the Fenton reactions are the major backbone for the degradation of organic substrates.^19^ Nickel ferrite (NiFe_2_O_4_), an inverse spinel ferrite, is a biocompatible, non-toxic, and ferrimagnetic material that is easy to prepare and very stable at ambient conditions.^23^ The composition of Fe^3+^ and Ni^2+^ in the composite makes them prominently used for catalytic reactions. Nowadays, conducting polymers are applied in many diverse applications due to their semiconducting properties, compatible nature, and fast responses.^24,25^ Among the various conducting polymers, polyaniline (PANI) is the most widely studied conducting polymer due to its low cost of production, stability, high conductivity, and environmentally benign nature.^26,27^ Also, the dopant ions in the PANI structure offer favorable sites for transferring the electrons to biomolecules and can accelerate the electron transfer between an electrode surface and electroactive molecules.^24,28,29^

Herein, we report the efficacy of the composite NiFe_2_O_4_–PANI (NF–PANI) for the sensing of AA by two orthogonal methods, namely as a peroxidase mimic and in electrochemical sensing. Though electrochemical methods are accurate and sensitive, colorimetric methods are preferable for a fast and cost-effective analysis. Thus, combining both advantages of colorimetric and electrochemical sensing is desired but still remains a challenge in the field of sensors. The present work elaborates the formations of nanocomposites of metal oxides and conducting polymers for use in colorimetric and electrochemical methods for sensing AA.

## 2. Experimental methods

### 2.1 Materials

The reagents hydrochloric acid (HCl), nickel chloride hexahydrate (NiCl_2_·6H_2_O), ferric chloride hexahydrate (FeCl_3_·6H_2_O), hydrogen peroxide (H_2_O_2_), urea, glucose, fructose, glycine, aspartic acid, 3,5,3,5-tetramethylbenzidine (TMB), ammonium persulfate (APS), aniline, l-cystine, and also the chemicals for the phosphate and sodium buffer preparations were procured from Himedia (P) Ltd., India. Commercially available orange juices were used for the real sample analyses. All the reagents were used as received without any further purification.

### 2.2 Synthesis of NF nanoparticles

First, 0.3244 g (0.1 M) ferric chloride was dissolved in 20 mL distilled water, and 0.356 g (0.05 M) nickel chloride was dissolved in 20 mL of distilled water separately and stirred for 20 min. The above solutions were mixed and 0.0567 g (0.1 M) of sodium borohydride was dissolved in 50 mL of water and added slowly into the mixture, which was stirred for another 2 h. The homogeneous mixture was transferred to the stainless steel autoclave and heated at 180 °C for 12 h. Further, the autoclave was allowed to cool naturally, and the precipitate was filtered. The obtained sample was washed several times with distilled water and finally with ethanol and then dried at 60 °C. The obtained nanoparticles were well ground and then calcined at 500 °C for 4 h. The final product was again well ground and used for further analysis.

### 2.3 Synthesis of NF–PANI nanocomposites

The NF–PANI nanocomposites were prepared by an *in situ* polymerization with as-prepared NF nanoparticles obtained through a hydrothermal process. Typically, 0.1 M of monomer aniline was initially dispersed in 25 mL of 0.1 M HCl dopant solution. Next, 2 g of as-prepared NF nanoparticles were added to the above solution, which was then sonicated to get a uniform dispersion. Simultaneously, the solution was cooled to an ice-cold condition between 0 °C to 5 °C. To the above mixture of monomer and nanoparticles, 0.15 M of APS prepared in 25 mL of 1 M HCl was added slowly and the mixture was then sonicated while maintaining the temperature below 5 °C. The mixture was left overnight and allowed to settle. A dark green precipitate was obtained, which was then washed several times with water and finally with methanol to remove the unreacted Cl ions present in the solution. The filtered product was dried below 70 °C without any further modification. PANI was synthesized by a polymerization method using the same process as the earlier described one without the addition of NF to the monomer solution.

### 2.4 Peroxidase mimic sensing

The catalytic activity of NF–PANI was evaluated by the colorimetric method using the chromogenic substrate TMB in the presence of H_2_O_2_. The reactions were carried out in sodium acetate buffer optimized at pH 3.5. Here, 100 μL of a fixed concentration of TMB was initially added to 2 mL of the buffer followed by the addition of 100 μL of a 10 mg/10 mL sample and a fixed concentration of H_2_O_2_, respectively. The solution was shaken well and allowed to react for 5 min. Then, the kinetic measurements for all the reactions were monitored in time course mode at 652 nm. The apparent kinetic parameters were estimated by using the Michaelis–Menten equation: 1/*ν* = (*K*_m_/*V*_max_)(1/[S]) + 1/*V*_max_, where *ν* is the initial velocity, *V*_max_ is the maximum intensity denoting the velocity, [S] is the concentration of the substrate, and *K*_m_ is the Michaelis constant.^19,30^ All the studies were carried out with the same procedure with varying the concentrations of the variant of interest.

### 2.5 Ascorbic acid sensing

Ascorbic acid detection was carried out with the same buffer with fixed concentrations of catalyst, TMB, and H_2_O_2_. Typically, after 5 min of adding the reactants, catalyst, TMB, and H_2_O_2_, 25 μL of AA with different concentrations was added to the reaction liquid. Finally, the catalytic kinetics was investigated for each concentration. Several potentially interfering compounds, such as urea, glucose, fructose, sucrose, starch, glycine, aspartic acid, dopamine, and cysteine were also prepared in the same concentration as that of AA and subjected to peroxidase mimic activity. The selectivity of the catalyst was examined through repeating the peroxidase activity with the interfering compounds separately and along with the presence of AA. To investigate the efficacy of the catalyst for sensing AA, we used commercial beverages and natural fruit juices. The orange juice (Minute Maid) and vitamin C tablets containing AA were procured from the open market and extracts from oranges and lemons were freshly prepared in the lab. The extract and commercial juices were diluted for obtaining various concentrations before analysis. The tablet was crushed and diluted for obtaining different concentrations. All the dilutions were made in the buffer solution.

### 2.6 Electrochemical sensing

The electrochemical investigations were performed through a three-electrode cell assembly. All the measurements, such as cyclic voltammetry (CV), chronoamperometry (CA), and differential pulse voltammetry (DPV) were carried out using a PAR analytical instruments, USA. A glassy carbon electrode (GCE) was employed as the working electrode and Ag/AgCl (3 M KCl) and Pt wire were employed as the reference and counter electrodes, respectively. The studies were performed by modifying the GCE using the NF–PANI nanocomposite as an electrocatalyst. GCE was cleaned with various grades of alumina (0.3 micron and 1 micron) prior to the modification. Here, 1 mg of NF–PANI nanocomposite was dispersed in a 2 mL ethanol + 5 μL Nafion mixture and 10 μL of the sample was drop-cast on the surface of GCE and dried overnight before analysis.

## 3. Results and discussions

### 3.1 Formation mechanism

**Scheme 1:**
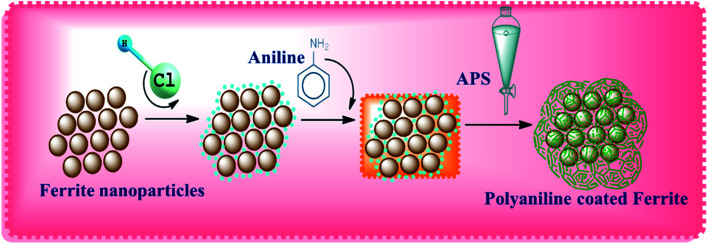
Formation mechanism of the NF–PANI nanocomposite.

Nickel ferrite consists of Fe ions in its outer structure, which makes the compound positively charged in 1 M of HCl. Generally, metal oxides possess a positive surface charge when the pH is below the point of zero charge (PZC), while they become negative above the PZC. The surface of magnetite has its PZC at pH 6, so it is positively charged in the monomer solution when it contains more protons than the hydroxyl groups. Therefore, Cl^−^ present in the solution gets absorbed on the surface of the NF nanoparticles and compensates the positive charge with ferrite ions. Thus, in the acidic solution, aniline monomers get converted to cationic anilinium ions. Thereby, electrostatic interactions occur between anionic Cl^−^ adsorbed on the nanoparticle's surface and cationic anilinium ions in the solution. The APS added into the mixture initiates the polymerization of the monomers on the nanoparticle's surface. This makes the composite nanostructure NF–PANI. A schematic illustration of the polymerization process of the nanocomposite is shown in Scheme 1.^31^

### 3.2 Characterization results and discussions

**Fig. 1:**
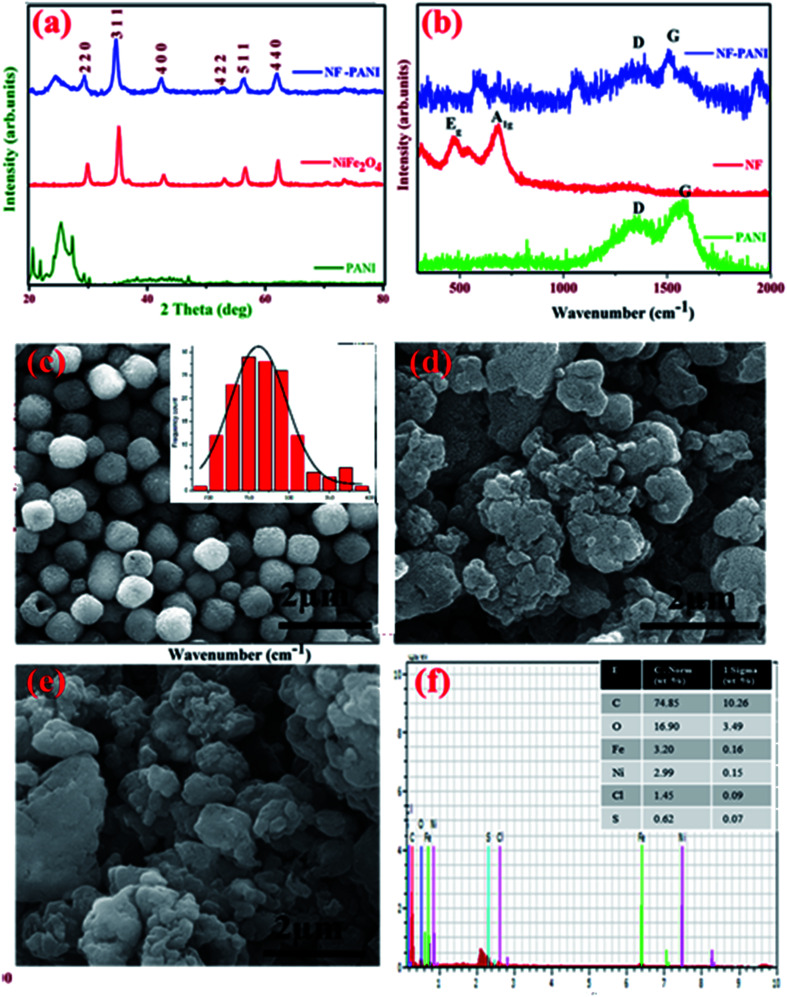
Structural, morphological, and elemental analyses of NF–PANI. (a) XRD analysis of pristine PANI, NF, and the composite NF–PANI; (b) Raman spectra of PANI, NF, and the composite NF–PANI; FESEM analysis of (c) NF, (d) PANI, (e) NF–PANI; and (f) elemental analysis of NF–PANI; with the inset table showing the % of elements present in NF–PANI.

The X-ray diffraction (XRD) pattern of PANI, NF, and the NF–PANI nanocomposite are shown in Fig. 1(a). The XRD peaks correspond to the typical inverse spinel structure (JCPDS # 10-0325) with the *Fd*3̄*m* space group.^32^ The average crystallite size was found to be 20 nm using the Scherrer formula. The polyaniline peak was obtained between 20–30° and the composite material also showed the peak at the same angle.^24^ There were no other impurity peaks observed in the XRD pattern that confirmed the formation of the amorphous form with the partially crystalline PANI. Instead, the XRD patterns of the nanocomposite NF–PANI showed all the major peaks of nickel ferrite with a hint of the formation of PANI with the XRD peak at 25°. This clearly depicted that the composite formation does not destroy the spinel structure of NF as well as the amorphous nature of PANI.

Raman spectroscopic analysis was carried out to strengthen the structural properties of the samples. Fig. 1(b) shows the Raman spectra for pure NF, PANI, and the NF–PANI nanocomposite. In pure nickel ferrite, only the E_g_ and A_1g_ modes of vibrations at 473 and 692 cm^−1^ could be observed.^33^ In PANI and the NF–PANI nanocomposites, the defective and graphitic peaks were present at 1300 and 1560 cm^−1^, which are the characteristic peaks of the C=C bonds of quinonoid and benzenoid units of PANI and the sp^2^ hybridized carbon atoms of carbon present in the polymer. The E_g_ and A_1g_ peaks were diminished in composites due to the composite formation. This may be due to the polymer formed over the surface of NF and the polymer matrix completely covering the surface of the ferrite. There was a significant change in the intensity of the D and G bands of the composites, which was due to the composite formation.

Fig. 1(c)–(e) show the field emission scanning electron microscopy (FESEM) images of NF, PANI, and the NF–PANI nanocomposites. Fig. 1(c) shows the hydrothermally synthesized nickel ferrite nanoparticles, which were spherical in shape with an average diameter ranging from 200–250 nm. The NaBH_4_ added in the reaction mixture acted as a reducing agent as well as a stabilizing agent and facilitated the Ostwald ripening process. Fig. 1(d) shows the FESEM micrographs of pure polyaniline in an aggregated form. The traditional oxidative chemical polymerization route is known to yield granular polyaniline.^34^Fig. 1(e) shows the morphology of nickel ferrite covered with polyaniline. The aniline polymerization takes place in the presence of nickel ferrite nanoparticles. Hence, the polyaniline nanostructures take nickel ferrite as an active nucleation point and cover their surface during polymerization. Thus, nickel ferrite particles could not be seen on the surface of polyaniline.^31^ The elemental analysis of the NF–PANI is shown Fig. 1(f), with the inset showing the atomic percentage of elements present in the sample. The EDAX results confirmed that the composite consisted of C, O, Fe, Ni, and miniscule percentages of S and Cl. The S and Cl were due to the leftover ions from HCl and APS after the reaction. No other impurity was present in the composite, which confirmed the elemental purity of the sample.

## 4. Sensing analysis of H_2_O_2_

**Fig. 2:**
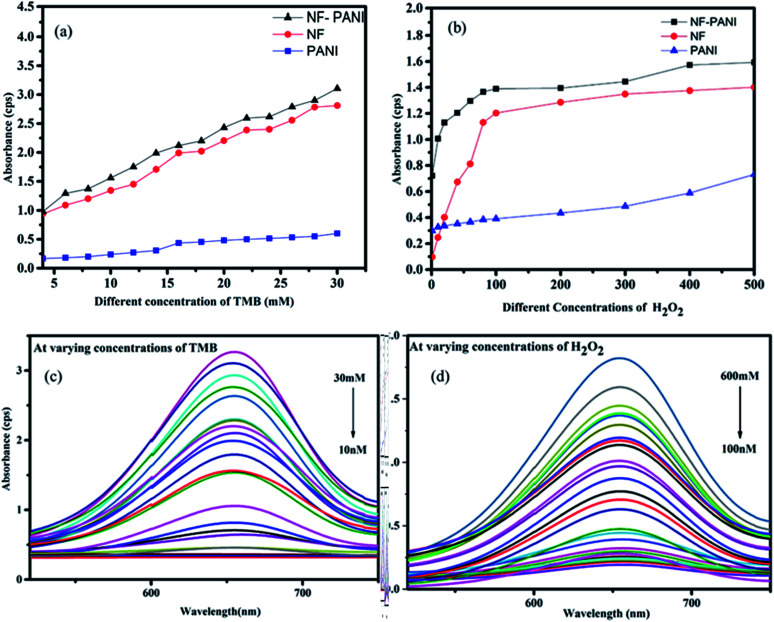
(a) Comparison of the catalytic activity upon varying the concentration of TMB, (b) comparison of the catalytic activity of all the samples at varying concentrations of H_2_O_2_, (c) change in the intensity of absorbance upon varying the concentration of TMB with the NF–PANI sample, and (d) intensity variations with the increase in the concentration of H_2_O_2_.

To investigate the peroxidase mimic activity of NF–PANI and to colorimetrically detect H_2_O_2_, TMB was used as a chromogenic substrate. It oxidizes in the presence of NF–PANI as a catalyst to change the colorless solution to a blue color. When the redox catalytic reaction occurred in the solution, it could be observed by the naked-eye and qualitatively analyzed by its UV-Visible absorbance spectrum. The reactions were carried out by adding fixed concentration of TMB in the acetate buffer (pH 3.5 optimized) of the required volume followed by the addition of the catalyst and varying concentrations of H_2_O_2_. Similarly, the sensing studies were repeated by varying the concentration of TMB with fixed concentrations of H_2_O_2_. The UV-Visible (UV) absorption spectrum of the solution after 10 min of reaction was recorded. The catalytic activity of NF, PANI, and NF–PANI were separately analyzed. Initially, the concentration of TMB was varied from 10 nM to 30 mM and the concentration of H_2_O_2_ was kept constant at 10 mM. The sample concentration was also kept constant as 100 μL from a 10 mg/10 mL dilution. The results confirmed that the NF–PANI composite had better activity than PANI and NF, as shown in Fig. 2(a) and (b), when varying the concentrations of both TMB and H_2_O_2_. The PANI samples showed very poor redox behavior in both the analyses; whereas the addition of NF on the PANI matrix enormously increased the peroxidase activity, which was due to the catalytic activity of Ni and Fe ions in the ferrite. Normally, in ferrites, their peroxidase enzyme mimic activity is ascribed to the Fenton reactions taking place in the solution. The H_2_O_2_ adsorbed on the nanoparticles is easily reduced by the electrons donated by the Fe^3+^ ions in the structure. The OH radicals are produced in either cases when Fe^3+^ or Fe^2+^ ion reacts with H_2_O_2_.^35,36^ This makes the composite a better catalytic agent in the peroxidase mimic sensing of H_2_O_2_.

The catalytic activity of NF–PANI at varying concentrations of TMB (30, 20, 10, 5, 1 mM, and 750, 500, 250, 100, 90, 80, 70, 60, 50, 40, 30, 20, 10 nM) and H_2_O_2_ (600, 550, 500, 450, 400, 350, 300, 250, 200, 150, 100, 50, 25, 10, 5, 1 mM, and 900, 800, 700, 600, 500, 400, 300, 200, 100 nM) is shown in Fig. 2(c) and (d) and was used to determine the linearity in the sensing and the limit of detection (LoD) of the sample. There was a linear increase in the intensity of absorbance with the increase in the concentrations of TMB and H_2_O_2_.

**Fig. 3:**
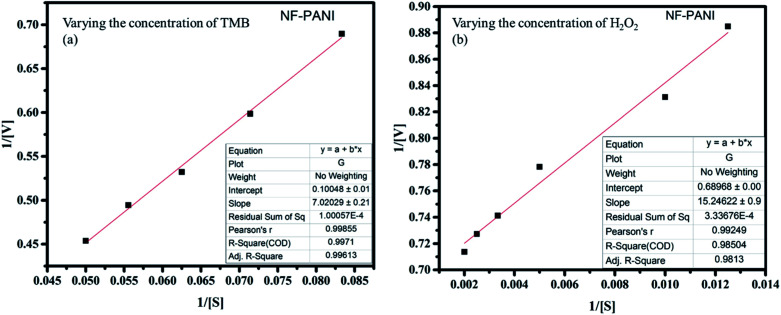
Linear peak fitting for the catalytic analysis with varying concentrations of (a) TMB and (b) H_2_O_2_.

The kinetic analysis of the catalytic redox reactions by the nanocomposite was further performed by linear fitting of the concentration-dependent variation in the absorbance spectrum. Fig. 3(a) and (b) show the linearly fitted graphs and their insets show the fitted results with the *R*^2^ values of 0.9961 and 0.9813 for TMB and H_2_O_2_, respectively. The rate of the reactions *V*_max_ and the Michaelis–Menten constant *K*_m_ were determined for NF, PANI, and NF–PANI and the values are given in Table 1, calculated utilizing the same calculation mentioned in our earlier work report. The *K*_m_ value indicates the enzyme affinity, where usually a lower *K*_m_ value indicates a higher affinity toward the substrate. Also, *V*_max_ represents the velocity of the reaction.^18^ The fitted parameters confirmed that the NF–PANI showed a higher affinity toward the substrate with a high *V*_max_. The kinetic studies clearly confirmed that the composite showed a better sensing ability for the sensing of H_2_O_2_. Similarly, the comparative activities of the catalysts with other sensing systems in the existing literature are also given in Table 2. The LoD of TMB was found to be 10 nM and 132 nM for H_2_O_2_. The lower LoD proves that the catalytic activity was efficient with even lower concentrations of TMB and H_2_O_2_.

**Table 1:** Kinetic analysis parameters for NF, PANI, and NF–PANI

Catalyst	Substance	*K*_m_ [mM]	*V*_max_ [10^−8^ M s^−1^]
NiFe_2_O_4_	TMB	39.7931	7.662
	H_2_O_2_	22.1265	1.4513
PANI	TMB	69.859	0.7092
	H_2_O_2_	36.859	0.5432
NiFe_2_O_4_/PANI	TMB	13.5045	9.9512
	H_2_O_2_	5.898	1.4684

**Table 2:** Comparison of the LoDs with the existing systems in the literature for sensing H_2_O_2_

Catalyst	Method	Limit of detection of H_2_O_2_	Reference
Fe_3_O_4_/CoFe–LDH hybrid	Colorimetric method	0.2 μM	33
Polyallylamine–IrO_2_/GO	Colorimetric method	324 nM	34
Fe^2+^, Co^2+^ and Ni^2+^	Electrochemical method	7.3 μM	37
HTCPP–ZnS	Colorimetric method	0.01 mM	38
NGZF	Colorimetric method	0.025 mM	19
NF–PANI	Colorimetric method	132 nM	Present work

## 5. Sensing of ascorbic acid

**Fig. 4:**
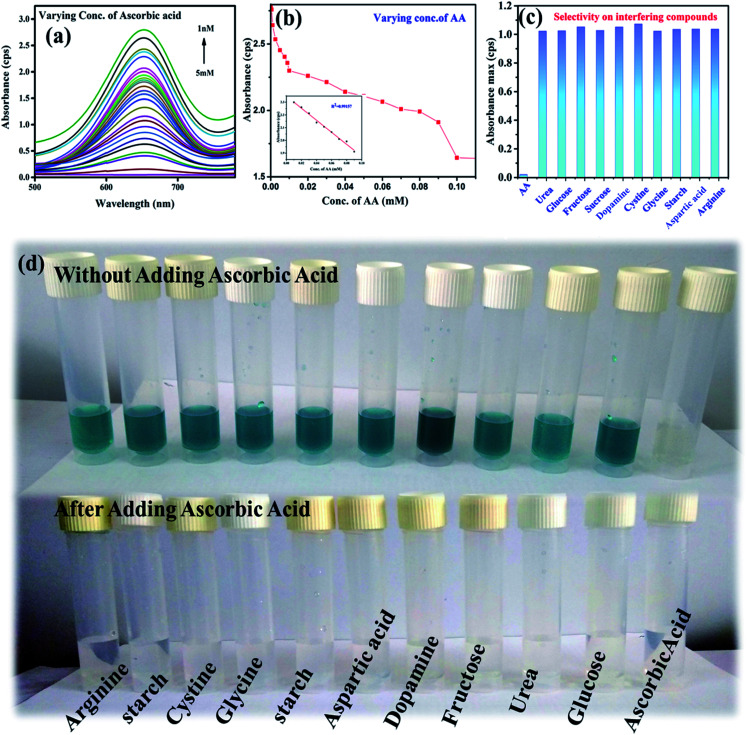
Ascorbic acid sensing (a) and (b) absorbance decrease with the increase in the concentration of AA, (b) inset shows the linearity with increasing the concentration of AA, (c) selectivity of the sensing of AA in the presence of potentially interfering compounds compared with AA at a 10 mM concentration each and (d) change in color with the addition of AA and with other interfering compounds with and without adding AA.

The sensing of AA was carried out by utilizing the peroxidase mimic property of the nanozyme. At optimal conditions, the sensing of AA was carried out using the NaAc buffer of pH 3.5. Fig. 4(a) and (b) show the gradual decrease in absorbance intensity with the increase in the concentrations of AA. The inset in Fig. 4(b) shows the linear relationship within the range of 10–100 μM for the absorbance and the concentrations of AA with the value of *R*^2^ = 0.99501 and the LoD = 232 nM through the relationship 3*σ*(standard deviation)/slope. This clearly shows that the catalyst was efficient for detecting AA to a lower range compared to the other reported values detected by other systems.^39,40^ In the peroxidase mimic sensing of H_2_O_2_, TMB oxidizes to produce a blue colored solution, whereas in AA sensing, when AA is added to the reaction solution, TMB reduces and oxidizes AA to become colorless. The increase in the concentration of AA decreases the color of the solution, which becomes transparent. This also suggests that AA forms a complex with the TMB substrate. It has already been reported that the substrate containing a nitrogen center has strong affinity toward AA,^1,41^ whereby it forms a strong covalent bond with nitrogen. In our study, the possible binding centers were terminal amine groups (–NH_2_) of the two TMB substrates. Thus, there was a decreasing intensity of absorbance with the increase in the concentration of AA in the reacting solution. Thus the number of effective TMB molecules to be oxidized by H_2_O_2_ in the presence of the catalyst became less. Further, the selectivity toward AA sensing was investigated by testing in the presence of potentially interfering compounds under optimized conditions. About 5 mM concentrations of various interferences, such as urea, glucose, fructose, sucrose, dopamine, starch, arginine, aspartic acid, cysteine, and glycine were considered.^42^ The same reaction conditions were maintained as for 5 mM of AA. There was no color change in the TMB solution. Further, when AA was introduced to each interfering solution mixture, the blue color of the TMB disappeared. Fig. 4(d) shows the changes in color with and without the addition of AA. The sensing ability was also compared with other existing literature reports and the results are given in Table 3. The sensitivity of the sample reached the nM concentration level, whereas the reported ones showed LoDs in the μM concentration range. Thus, the present work emphasizes the sensing of H_2_O_2_ to a very low concentration, which may be applicable for biological applications.

**Table 3:** Comparison of the LoD with systems in the existing literatures for the sensing of AA

Catalyst	Method	Limit of detection of ascorbic acid	Reference
Gr/CuPc/PANI	Electrochemical method	6.3 × 10^−8^ M	7
PANI/HNTs	Electrophoretic deposition method	0.21 μM	39
Graphene-based 3D nanocomposites	Electrochemical method	460 μM	40
Polymer-coated electrodes	Electrochemical method	0.0267 μM	43
PANI/MnO_2_–Sb_2_O_3_	Electrochemical method	0.12 mM	44
S,N co-doped graphene quantum	Fluorescence method	1.2 μM	45
Carbon dots on CoOOH	Fluorescence method	25 nM	46
MoS_2_-decorated N-doped carbon nanotubes	Colorimetric method	0.12 mM	47
Nickel ferrite nanoparticles on a carbonaceous matrix	Colorimetric method	260 nM	48
Mustard seeds	Colorimetric method	3.26 μM	49
3,4:9,10-perylene tetracarboxylic acid-modified zinc ferrite	Colorimetric method	0.834 μM	50
PANI–MnO_2_	Colorimetric method	26 nM	29
NF–PANI	Colorimetric method	0.232 nM	Present work

### 5.1 Real sample analysis

The practical viability of the present AA sensor was also investigated with real samples. Orange and lemon extracts and vitamin C tablet were diluted to three different concentrations (25, 50, and 100 μM) for the present investigation.^1,51^ As listed in Table 4, the recoveries of a known amount of AA in the 500-fold diluted real samples were between 97.5% and 106.97% with the RSD ranging from 1.7% to 3.1%. The results clearly indicated that the proposed sensor is applicable for the quantification of AA in natural as well as in commercial AA-containing foods and beverages.^52^

**Table 4:** Real sample analysis for the sensing of AA present in fresh and commercially available fruit juices and tablets

Sample	Added (μM)	Found (μM)	Recovery (%)	RSD (%, *n* = 3)
Orange juice	25	24.37	97.48	2.8
	50	50.12	100.24	3.1
	100	103.56	103.56	2.6
Lemon extract	25	26.24	106.97	2.2
	50	48.89	97.78	1.7
	100	101.84	101.84	1.9
Vitamin C	25	25.34	97.36	2.1
	50	49.35	98.7	1.9
	100	98.61	98.61	2.3

## 6. Electrochemical sensing of AA

### 6.1 Electro-oxidation of AA by NF–PANI-modified GCE

**Fig. 5:**
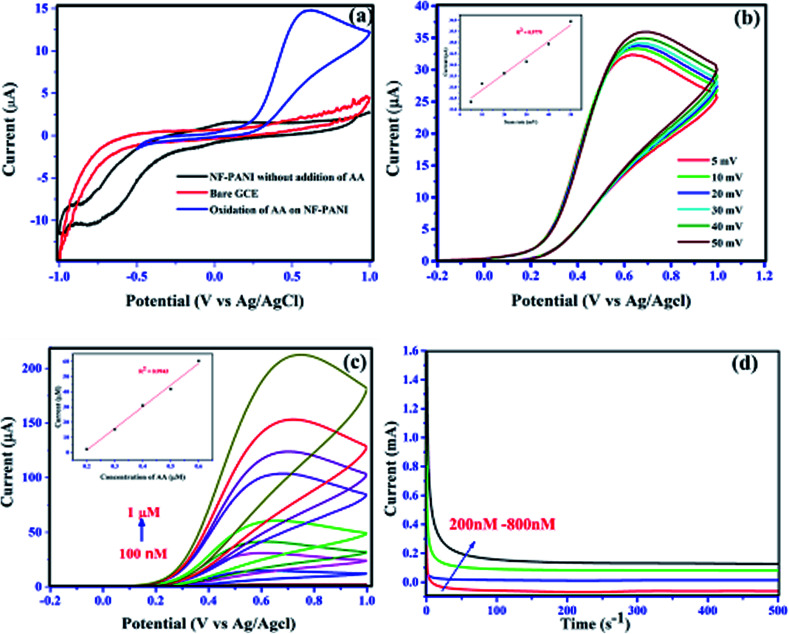
Electrochemical oxidation of ascorbic acid: (a) CV analysis of bare GCE, and NF–PANI-modified GCE with and without the addition of AA, (b) CV analysis of NF–PANI at different scan rates for the detection of AA; inset shows a linear fit of the scan rate, (c) voltammograms showing the increase in peak current with the increase in concentration of AA; inset shows its linear fit, and (d) chronoamperometric graph of different concentrations of AA at a fixed potential of 602 mV.

**Scheme 2:**
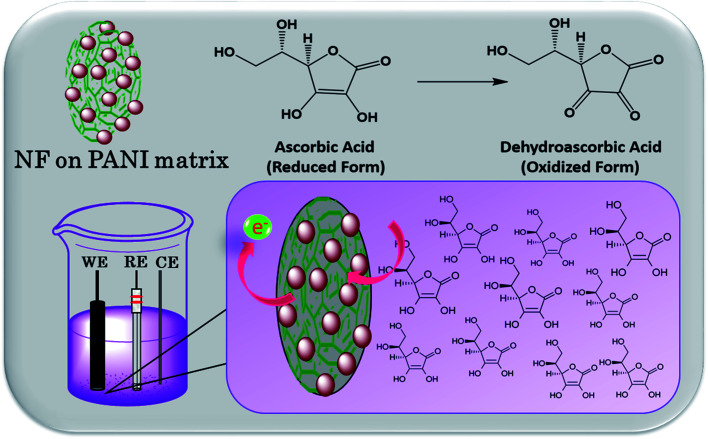
Schematic illustration for the electrochemical sensing of ascorbic acid.

The electrochemical sensing of AA was performed by analyzing the oxidative property of the nanocomposite. Herein, we investigated the redox capability of AA on the surface of NF–PANI with the help of cyclic voltammetry (CV), chronoamperometry (CA), and differential pulse voltammetry (DPV). All the measurements were performed at various pH values and it was found that the activity was high at pH 7 of PBS, which was chosen as the optimized pH for further studies. Fig. 5(a) shows the cyclic voltammograms of bare GCE and NF–PANI in PBS compared with the NF–PANI upon the addition of 500 nM of AA. It can be clearly seen that the bare GCE and NF–PANI did not show any oxidation or reduction peaks before the addition of AA, whereas upon the addition of a fixed concentration of AA to the reaction solution, an oxidation peak at 602 mV corresponding to the change of AA from dehydroascorbic acid could be observed. To strengthen the analysis of the oxidative property of NF–PANI toward AA, scan rate and concentration-dependent analyses were performed. Fig. 5(b) shows the increase in peak current in CV for different scan rates with a fixed concentration of AA, which confirmed the influence of the catalytic activity of the NF–PANI nanocomposite.^3,4^ The inset of Fig. 5(b) shows the linear fitting of the peak currents at different scan rates of the CV. The sensing tests of AA with varying concentrations from 100 nm to 1 μM were also performed with CV, CA, and DPV. The voltammograms of NF–PANI with varying the concentrations of AA are shown in Fig. 5(c). The peak current was increased by increasing the concentrations of AA, which clearly confirmed that the catalyst improved the oxidation of incoming AA in contact with the catalyst. The linearity of the sensing was analyzed by linear fitting the data between 200–1000 nM and this is shown in an inset graph with *R*^2^ = 0.9943. Fig. 5(d) shows the chronoamperometric investigation with varying the concentrations of AA with the fixed potential of 602 mV, showing that the catalyst maintained a stable oxidation current for all the concentrations. This suggests that the NF–PANI nanocomposite showed excellent sensing performances.^26,53^ A schematic illustration for the sensing of AA by the electro-oxidation of NF–PANI in PBS is shown in Scheme 2.

**Fig. 6:**
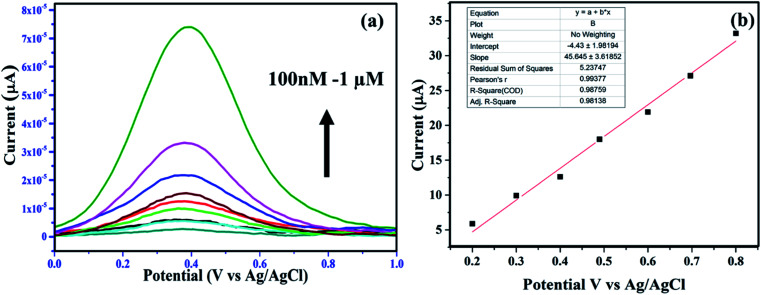
(a) DPV analysis of NF–PANI-modified GCE; the peak current increases with the increasing concentration of AA and (b) linear fitting of the concentration-dependent increase in the peak current with *R*^2^ = 0.9875.

The peak current of ascorbic acid oxidation at the surface of the NF–PANI can be used to detect the concentration of ascorbic acid. To further study the catalytic property of NF–PANI, DPV analysis of the electrode was carried out and the results are shown in Fig. 6(a). The oxidation of AA occurred at 0.401 V, where the peak current increased with the concentration of AA added.^54^ The fitting of the peak current gave a straight line, as shown in Fig. 6(b). The linear fitting gave an *R*^2^ value of 0.98759, which suggests the linearity of the sensing. The oxidation peak currents of ascorbic acid at the electrode surface were proportional to the concentration of the ascorbic acid in the range of 0.1 to 1 μM. The detection limit (3*σ*) of ascorbic acid was found to be 0.423 × 10^−6^ M.^43,53^

### 6.2 Repeatability and stability of the nanocomposite

The long-term stability of the NF–PANI nanocomposite was established through analysis of the catalyst over a 7 day period. The modified electrode was stored for a week at room temperature without any further modification and the experiments were performed again. According to the voltammograms, there was no change in the peak potential for ascorbic acid oxidation, whereas the intensity of the peaks decreased by 1.2% compared with the initial response. This may be ascribed to the ageing effect of the electrodes. Similarly, the repeatability of the samples was studied by preparing the electrode three times and analyzing separately, and the results are attached in the ESI 1.† According to the results, the NF–PANI-modified electrodes had increased sensitivity and stability toward the oxidation of AA.

## 7. Conclusions

In summary, a nanocomposite NF/PANI with excellent peroxidase mimic activity and electrochemical behavior toward the sensing of AA was reported. The composite nanosensor combining the influence of the Fenton reactions of NF and the high surface area of PANI's conductive network provided excellent catalytic performance in both sensing methods, electrochemical and colorimetric. Also, the AA present in the real samples was detected with an RSD ranging from 1.7% to 3.1%. Moreover, the samples showed good selectivity toward the interfering compounds with an LoD of 48 nM. Similarly, the electrochemical studies revealed the sensing ability of AA with high accuracy, and the LoD of this method was found to be 423 nM. Though the compound showed a low limit of detection in the peroxidase method, the electrochemical activity also showed comparable results with existing electrochemical sensors. Thus, the nanocomposite has the potential to mimic peroxidase in the sensing of chemical compounds as well as can act as an efficient electrochemical sensor in selectively detecting AA. The obtained results suggest that the nanocomposite can be utilized as a real-time sensor for instant lab analysis and also as an electrochemical sensor. This will invoke new attempts by researchers to study the dual property of the material to develop novel duo-sensors.

## Conflicts of interest

There are no conflicts to declare.

## Supplementary Material

NA-002-D0NA00283F-s001
