# Supplementary material for: A nanocomposite of NiFe2O4–PANI as a duo active electrocatalyst toward the sensitive colorimetric and electrochemical sensing of ascorbic acid
Source: Nanoscale Adv. 2020 Jun 22;2(8):3481–93. doi: 10.1039/d0na00283f (PMC9417939; doi:10.1039/d0na00283f)
Supplement: NA-002-D0NA00283F-s001 [file NA-002-D0NA00283F-s001.pdf]

Electronic Supplementary Material (ESI) for Nanoscale Advances.
This journal is © The Royal Society of Chemistry 2020

Electronic Supplementary Information

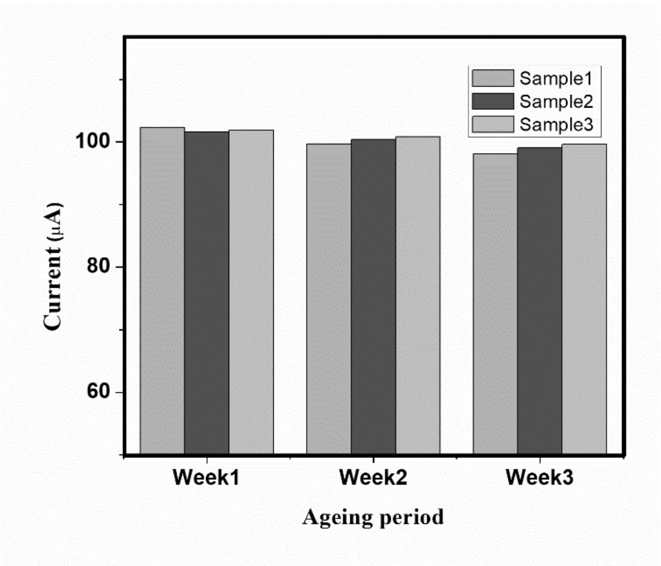

SI.1: Repeatability and Reproducibility of the sample carried out with three different electrodes prepared with the same sample denoted as sample 1, 2 and 3. The electrode was aged for three weeks and every week the analysis is carried out in all three samples
